# Medical Diagnosis with Special Reference to Practical Medicine

**Published:** 1876-02

**Authors:** 


### Bibliographical.

---

MEDICAL DIAGNOSIS WITH SPECIAL REFERENCE TO PRACTICAL MEDICINE. A guide to the knowledge and discrimination of disease. By J. M. DaCosta, M.D., Professor of the Practice of Medicine and of Clinical Medicine, at the Jefferson Medical College, Philadelphia; Physician to the Pennsylvania Hospital; Consulting Physician to the Children's Hospital; Fellow of the College of Physicians, etc. Illustrated with engravings on wood. Fourth edition, revised. Philadelphia, J. P. Lippincott & Co. 1876.

Since the first edition of this work, in 1864, it has been constantly gaining in public favor, so that the last two editions have been rapidly exhausted. In the fourth edition numerous additions and changes have been made, without, however, materially adding to the size of the work.

For advanced students and practitioners of medicine there is no work on medical diagnosis more valuable. The distinguished author, Dr. DaCosta, is too well known to the profession of America to require commendation at our hands. The work may be procured at publishers' prices at Phillips & Crews, Marietta street, Atlanta, Georgia.

